# Dopamine Modulates the Rest Period Length without Perturbation of Its Power Law Distribution in *Drosophila melanogaster*

**DOI:** 10.1371/journal.pone.0032007

**Published:** 2012-02-16

**Authors:** Taro Ueno, Naoki Masuda, Shoen Kume, Kazuhiko Kume

**Affiliations:** 1 Department of Stem Cell Biology, Institute of Embryology and Genetics, Kumamoto University, Kumamoto, Japan; 2 Graduate School of Information Science and Technology, the University of Tokyo, Tokyo, Japan; 3 PRESTO, Japan Science and Technology Agency, Kawaguchi, Saitama, Japan; 4 Global COE program, Kumamoto University, Kumamoto, Japan; University of California Los Angeles, United States of America

## Abstract

We analyzed the effects of dopamine signaling on the temporal organization of rest and activity in *Drosophila melanogaster*. Locomotor behaviors were recorded using a video-monitoring system, and the amounts of movements were quantified by using an image processing program. We, first, confirmed that rest bout durations followed long-tailed (i.e., power-law) distributions, whereas activity bout durations did not with a strict method described by Clauset *et al.* We also studied the effects of circadian rhythm and ambient temperature on rest bouts and activity bouts. The fraction of activity significantly increased during subjective day and at high temperature, but the power-law exponent of the rest bout distribution was not affected. The reduction in rest was realized by reduction in long rest bouts. The distribution of activity bouts did not change drastically under the above mentioned conditions. We then assessed the effects of dopamine. The distribution of rest bouts became less long-tailed and the time spent in activity significantly increased after the augmentation of dopamine signaling. Administration of a dopamine biosynthesis inhibitor yielded the opposite effects. However, the distribution of activity bouts did not contribute to the changes. These results suggest that the modulation of locomotor behavior by dopamine is predominantly controlled by changing the duration of rest bouts, rather than the duration of activity bouts.

## Introduction

Human behavior is highly diverse and profoundly complex. Our decisions are influenced by both our internal drive and our perception of environments. However, recent studies have shown that many human behaviors have shared common characteristics in the temporal organization. Although current models of human dynamics assume that human actions are randomly distributed in time and thus well approximated by Poisson processes, there is increasing evidence that the timing of many human activities follow non-Poisson statistics, characterized by bursts of rapidly occurring events separated by long periods of inactivity. Interevent intervals of social behaviors such as e-mail communications and trade transactions follow power-law distributions [1]. How rest and activity episodes are interwoven in behaving animals was also studied recently [2], [3], indicating that rest bouts follow the power-law distribution in both human and mice, whereas activity bouts follow the exponential distribution. Furthermore, Nakamura et al, reported that patients with major depressive disorder exhibited decrease of scaling exponent in power-law distribution of resting period, suggesting novel quantitative strategy for neuropsychiatry [3]. Although power-law distributions are abundant in various natural phenomena and thus do not indicate the universality of animal behaviors by themselves, the biological mechanism underlying the power law distributions attracts attentions and remains an open question.

Similar temporal organization of rest and activity bouts has also been observed in invertebrates. In an insect *Drosophila melanogaster*, waiting intervals between behavioral episodes such as walking, feeding, and flight maneuvers follow the power-law distribution [4], [5], [6], [7], [8]. A wide range of similarities have been reported between insects and mammals with respect to the genes that regulate behaviors such as clock genes regulating circadian rhythm and sleep-wake cycle related genes. We believe that understanding the temporal organization of behavior in *Drosophila melanogaster*, the most promising model organism for studying the molecular basis of behavior, would shed light on the biological mechanism underlying common behavioral characteristics. For the analysis of temporal organization, precise fitting method to power-law distribution is indispensable. The previous report drew attention to an issue that conventional methods such as simple linear fitting of data plotted on log-log axes tend to place overweight on large but rare events, thus favor the power-law distribution [9], [10]. Most of previous studies which showed the power-law distribution in fly locomotion used the linear regression method. Moreover, it was recently demonstrated that even when individual animals move in a predominantly diffusive manner, they may collectively display superdiffusive characteristics, often interpreted as a Lévy flight, i.e. a power law, due to the variation at population level [11]. Thus, proving a power law distribution at the individual animal level is critical. Here we applied modern statistical methods to test whether the temporal organization of *Drosophila* rest provides more evidence for a power-law distribution.

Despite the large number of studies on the locomotor activity of *Drosophila*, the technological constraints of the apparatus used for measuring locomotor activity have limited the quantitative understanding of its temporal organization. Most of the previous studies were conducted using an infrared beam apparatus such as the *Drosophila* activity monitoring (DAM) system (Trikinetics, Waltham, MA), which counts infrared beam crossings of flies and thus detects movements only when a fly crosses specific parts of the chamber. Because flies need to walk for some distance between crossings, the time resolution, which depends on the length of the chamber, is typically a few seconds (3–10 s). The spatial resolution of the recording is also limited by the distance between adjacent crossings determined by the length of the housing glass tube, which is about 5 cm. Although this system is suitable for studying circadian rhythm and observing gross changes in activities, its spatial and temporal resolutions are not adequate for revealing precise temporal architecture of rest and activity bouts. Furthermore, the DAM system overestimates rest bouts [12]. Previous studies on fly locomotor activity performed using a video method [13], [14], [15], [16], [17], which realizes high spatial and temporal precision, were restricted in terms of the observation period (2–7 h) due to the lack of fly's food.

For a precise analysis of behavioral organization in *Drosophila melanogaster*, we developed a video monitoring and movement analysis system. Using this system, we showed that, in *Drosophila*, the rest bouts follow the power-law distribution, whereas the activity bouts do not. Our results suggest that the temporal organization of rest and activity episodes in *Drosophila* shares common features with mammals [3].

We have worked on sleep in *Drosophila*, especially using a short sleeper mutant, *fumin*, which we discovered and demonstrated is a dopamine transporter mutant. And we and others identified dopamine as a regulator of sleep/wake cycling in *Drosophila* [18], [19]. Thus, we are interested in the way how dopamine regulates rest/activity, and we analyzed the effects of dopamine. We found that dopamine modulates the rest length without perturbation of the temporal organization of locomotor activity When the total amount of activity per day (and thus that of rest) is modulated, the modulation occurs through the changes in the number of activity episodes but not their length. On the other hand, the gross changes in rest are regulated by changing both the number and length of rest episodes.

## Results

### Power-law distribution of rest bouts

**Figure 1:**
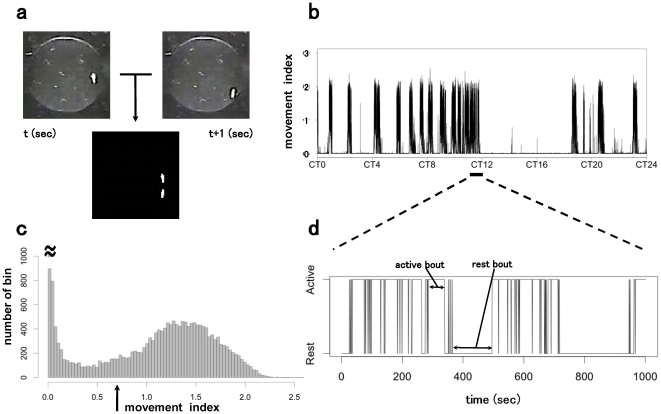
Movement quantification and data analysis. a. Amount of movement was determined by the quantification of the absolute difference between consecutive images. The two images in the top row represent a single fly in a chamber at two consecutive time points. The differential image (bottom) was generated by the subtraction of these two images followed by binarization. The degree of movement was defined as the percent of suprathreshold pixels in the total number of pixels in ROIs. b. 24-h recording of the degree of movement of a single fly. c. Histogram of the degree of movement. The arrow indicates the threshold determined by the *k*-means clustering algorithm. d. Expanded and binarized view of b. The duration of the trace shown in this panel is indicated by the horizontal bar in Figure 1b. Each bin was classified to rest or activity according to the thresholding of the degree of movement.

Figure 1b shows a typical time course of the movements of a control fly for 24 h. As shown in Figure 1c, the degree of the movement in each bin of 1-s width shows a bimodal distribution. Therefore, we classified the bins into rest and activity bins using the *k*-means clustering algorithm. We then determined the rest bouts and activity bouts. Figure 1d shows a series of alternating rest and activity episodes.

**Figure 2:**
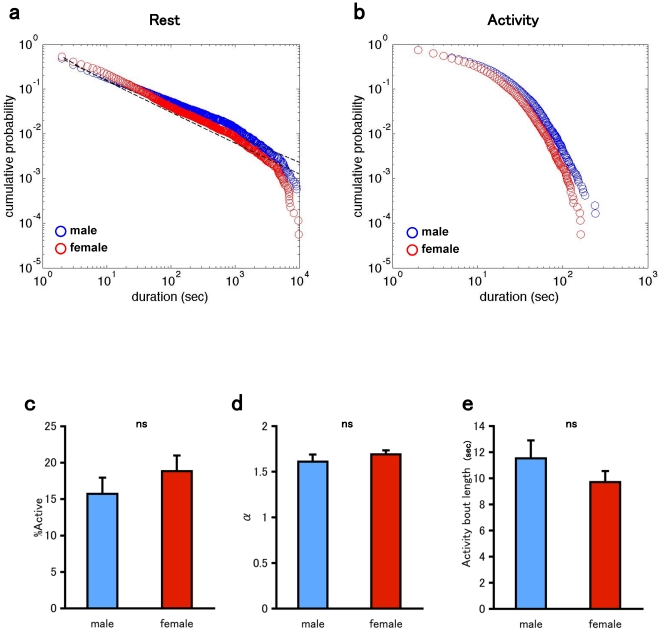
Distributions of rest and activity bouts during locomotor activities in *Drosophila.* a. Double logarithmic plots of the cumulative probability of the rest bout for the combined data of control flies (n = 10). blue: male. red: female. The broken line represents best fits to the data (see Materials and Methods for the fitting procedure). b. Double logarithmic plots of the cumulative probability of the activity bout for the combined data of control flies (n = 10). blue: male. red: female. c. The ratio of the total duration of the activity episodes. d. Power-law exponent of the fitted distribution of rest bouts. e. Mean activity bout length. Bars and error bars represent the mean and standard error of the mean (s.e.m.), respectively. ns, not significant (Student's *t* test).

We separately analyzed the temporal structure of rest and activity bouts. The cumulative distribution of rest bouts for control on the double logarithmic scale is shown in Figure 2a. The approximately linear region of the distribution nearly 3 decades indicates the power-law nature of the distribution of rest bouts. The solid line represents the best fit to the data determined by the maximal likelihood method [20]. In contrast, the activity bouts do not obey a power-law distribution (Figure 2b). We performed statistical analysis to determine the validity of the power-law fit (see Methods). The results are summarized in Table 1. The rest bout showed a significantly better fit to the power-law distribution than to the exponential distribution. However, the rest bout did not show better fit either to the power-law or to the log-normal distribution, when these two were compared. Clauset et al. described that ‘it is extremely difficult to tell the difference between log-normal and power-law behavior and it appears unlikely that any test would be able to tell them apart unless we had an extremely large data set’ [20]. On the other hand, the hypothesis of the power-law distribution for activity bouts was not supported as compared to the exponential distribution. In Figure 2, the data from different flies are combined because a single fly does not provide sufficient data points. However, the results obtained for the combined data (Figure 2) are reproduced for the data for single flies (Figure S1); they are not a byproduct of combining the data from different flies.

**Table 1:** Statistical tests for the significance of the power-law property in the distributions of rest and activity bouts according to the method of Clauset et al. [20].

Group	Rest					Activity				
	power-law	exponential		log-normal		power-law	exponential		log-normal	
	p′	LR	p	LR	p	p′	LR	p	LR	p
control	0.21	9.65	0.00	−1.17	0.37	0.23	0.02	0.54	−1.89	0.18
control female	0.32	8.43	0.00	−1.35	0.36	0.30	−0.87	0.35	−1.70	0.17
control Day	0.16	9.74	0.00	−0.93	0.30	0.29	0.29	0.49	−3.78	0.12
control Night	0.28	10.71	0.00	−2.09	0.18	0.30	−0.03	0.64	−2.84	0.24
control (20°C)	0.07	10.26	0.00	−1.69	0.25	0.24	−0.61	0.44	−1.55	0.18
control (30°C)	0.03	10.98	0.00	−1.74	0.18	0.16	−1.08	0.29	−2.06	0.08
*fmn*	0.16	8.83	0.00	−0.37	0.11	0.02	−2.69	0.19	−3.54	0.01
control + 3IY	0.20	8.28	0.00	0.31	0.35	0.08	1.09	0.32	−1.52	0.17
*fmn* + 3IY	0.34	8.78	0.00	0.69	0.22	0.23	0.88	0.29	−1.78	0.13
TH-GAL4; UAS-dTrpA1(22°C)	0.31	9.39	0.00	−1.00	0.33	0.07	−0.94	0.43	−2.94	0.14
TH-GAL4; UAS-dTrpA1(29°C)	0.12	4.30	0.00	−1.77	0.25	0.08	−1.42	0.30	−4.08	0.01

All the tests were separately conducted for the data obtained from individual flies. The mean statistical values for different flies are shown in the table. For each empirical distribution, we calculated the *p′-*value for the best power-law fit and likelihood ratios (LRs) of the power-law distribution to alternative distributions. We used the exponential distribution and the log-normal distribution as the alternative distributions. Positive values of the log likelihood ratio with p<0.05 indicate that the power-law distribution is statistically favored over the alternative distribution.

We set the size of the arena to 2.5 cm in diameter in these experiments. However, the distributions of rest and activity bouts did not change in either a bigger (5 cm in diameter) or a smaller (1 cm in diameter) arena (Figure S2).

Finally, for a later use, we calculated the ratio of activity (i.e. time spent in activity per observation period), the power-law exponent of the rest bout (Figure 2c,d). For statistical analysis of activity bouts which do not follow long-tail distribution, we calculated the mean activity bout length which allows comparison with conventional sleep study in *Drosophila* [21].

We also analyzed the data recorded from female flies. To exclude the interference with egg laying behavior, we used virgin females of control fly. The results for female flies are similar to those for male flies; rest bouts follow a power-law distribution, whereas activity bouts do not (Figure 2, Table 1) although sexual dimorphism of fly locomotion in mated female are reported [22], [23].

### Effects of circadian rhythm

**Figure 3:**
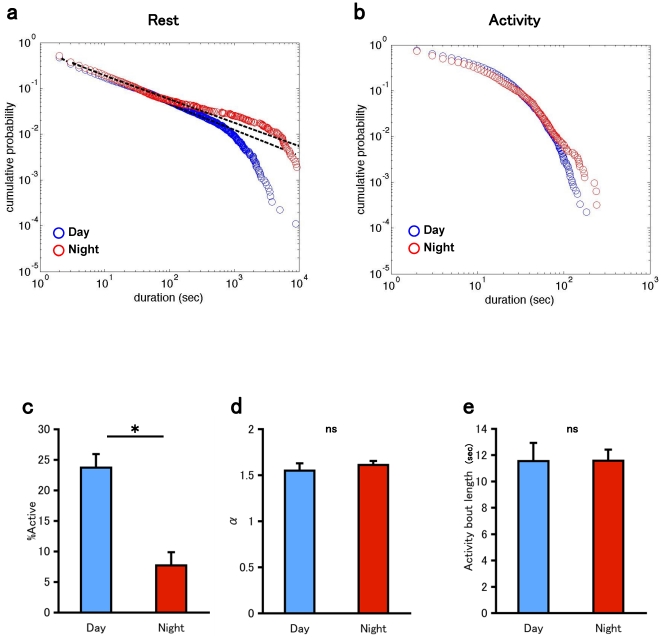
Effects of circadian rhythm. a. Double logarithmic plots of the cumulative probability of the rest bout for the combined data of control male flies (n = 10). blue: subjective day. red: subjective night. b. Double logarithmic plots of the cumulative probability of the activity bout for the combined data of control male flies (n = 10). blue: subjective day. red: subjective night. c. The ratio of the total duration of the activity episodes. d. Power-law exponent of the fitted distribution of rest bouts. e. Mean activity bout length. Bars and error bars represent the mean and s.e.m., respectively. Asterisk indicates statistically significant difference (p<0.05 Student's *t* test).

*Drosophila melanogaster* shows a diurnal activity pattern in an isolated experimental condition, although it is usually classified as a crepuscular organism. Owing to the circadian rhythm, the fly shows more locomotor activities during the subjective day than during the subjective night even under constant darkness, as shown in Figure 1b. To assess the effect of circadian rhythm on the temporal organization of rest and activity bouts, we separately analyzed the data from the subjective day and those from the subjective night. The ratio of activity in the subjective day was approximately 3-fold higher than that in the subjective night (Figure 3c). Nevertheless, the power-law distribution of rest bouts was observed in both subjective night and subjective day. The power-law exponents of the rest bouts were not significantly different between subjective day and night, and the difference was observed at the long rest bout around 1,000∼10,000 second (Figure 3a,d). The circadian modulation of rest was realized by the change in the frequency of long rest bouts, rather than in the power-law exponent of the distribution of rest bouts. On the other hand, the length of activity bouts was not drastically different between the subjective day and the subjective night (Figure 3b,e). We further observed that power-law property of rest bouts is sustained in rhythm mutant (Figure S3). These results suggest that power-law distribution of rest bouts is independent from circadian clock.

### Effect of temperature

**Figure 4:**
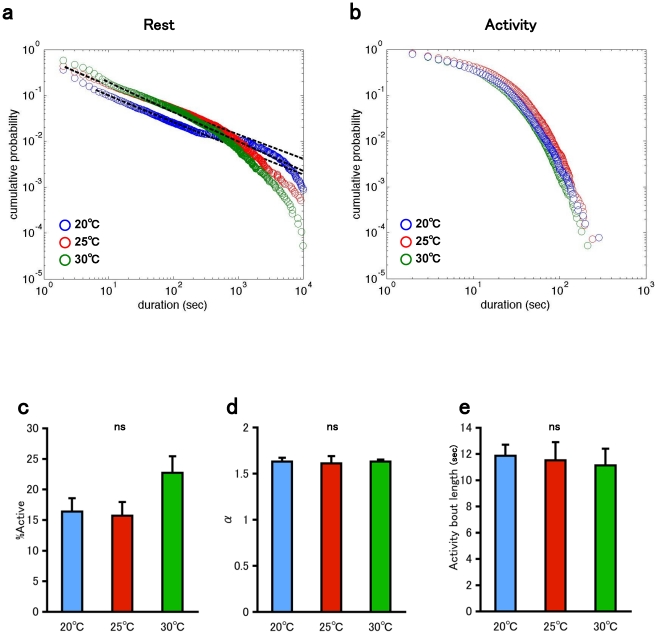
Effect of temperature. a. Double logarithmic plots of the cumulative probability of the rest bout for the combined data of control male flies (n = 10). blue: 20°C. red: 25°C. green: 30°C. b. Double logarithmic plots of the cumulative probability of the activity bout for the combined data of control male flies (n = 10). blue: 20°C. red: 25°C. green: 30°C. c. The ratio of the total duration of the activity episodes. d. Power-law exponent of the fitted distribution of rest bouts. e. Mean activity bout length. Bars and error bars represent the mean and s.e.m., respectively. ns, not significant (Tukey–Kramer HSD test).

Rates of many biological processes increase at high temperature. For example, many organisms show a shorter development time and shorter life span at higher temperature [24]. Thus, the ambient temperature is strongly associated with biological time although circadian rhythm shows temperature compensation. Ambient temperature also affects the locomotor activity, and the activity of a fly increases with temperature [25], [26]. To examine the effect of ambient temperature on the temporal organization of rest and activity episodes, we recorded the data at 20°C and 30°C for 24 h. The rest bout followed a power-law distribution, and the ratio of activity increased at 30°C (Figure 4a,c). On the other hand, the distribution of activity bouts did not change drastically with temperature (Figure 4d). Similar to the case of the circadian effect, the length of activity bouts was not drastically different between these temperature (Figure 4b,e).

### Effects of dopamine

**Figure 5:**
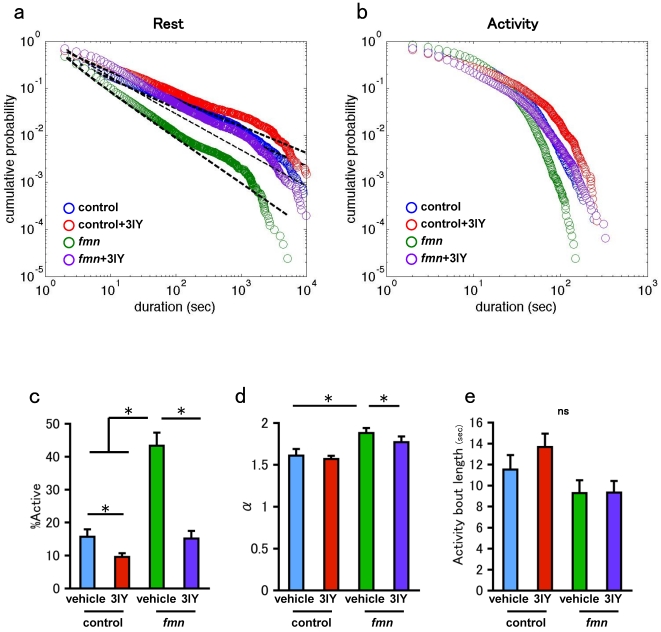
Effect of dopamine. a. Double logarithmic plots of the cumulative probability of the rest bout for the combined data of male flies (n = 10). blue: control. red: control fed with 3IY. green: *fmn*. purple: *fmn* fed with 3IY.*fmnfmn*. b. Double logarithmic plots of the cumulative probability of the activity bout for the combined data of control male flies (n = 10). blue: control. red: control fed with 3IY. green: *fmn*. purple: *fmn* fed with 3IY.*fmnfmn*. c. The ratio of the total duration of the activity episodes. d. Power-law exponent of the fitted distribution of rest bouts. e. Mean activity bout length. Bars and error bars represent the mean and s.e.m., respectively. Asterisks indicate statistically significant difference (p<0.05 Tukey–Kramer HSD test).

Dopamine is a neural transmitter that affects sleep and locomotor activities in both mammals and insects [18], [27]. Although the changes in the gross amount of sleep and activity episodes by dopamine signaling are well described, its effect on the temporal organization of rest and activity episodes is elusive. To address this issue, we used the *fumin* (*fmn*) mutant. *fmn* is the loss-of-function mutant of dopamine transporter. Dopamine transporters are expressed almost exclusively in the dopaminergic neurons [28] and regarded to function in the presynaptic membrane. They reuptake released dopamine and diminish dopamine signaling [29]. Thus, a mutation in this transporter results in the augmentation of dopamine signaling and causes an increase of the ratio in the duration of the active state, that is hyperactivity phenotype. Our video-monitoring system also showed that the *fmn* mutant flies displayed the hyperactivity phenotype. Interestingly, the power-law property of the distribution of rest bouts was maintained in *fmn* mutant flies (Figure 5a, Table 1). The ratio of activity and the power-law exponent for the rest bout was significantly larger in the *fmn* mutant flies than in the control flies (Figure 5c,d). On the other hand, the distribution of activity bouts did not differ drastically between the control and *fmn* mutant flies (Figure 5b,e). The hyperactivity of the *fmn* mutant is mainly realized by the modulation of the rest bout, and not by that of the activity bout.

In order to study the effect of dopamine in more detail, we exposed *fmn* flies to an inhibitor of dopamine biosynthesis, 3-iodo-tyrosine (3IY). 3IY inhibits the rate-limiting enzyme of dopamine biosynthesis, i.e., tyrosine hydroxylase, and significantly decreases the steady-state amount of dopamine after 2 days of consuming food mixed with 3IY [30]. The drug was mixed with the food provided to the flies, and the flies were exposed to the drug during the assay. As shown in Figure 5c, 3IY application decreased the ratio of activity as reported previously [19]. 3IY did not destroy the power-law property of the distribution of rest bouts, whereas it decreased the power-law exponent (Figure 5a,d Table 1). As a result, the ratio of activity decreased after the flies were exposed to 3IY. 3IY did not change the distribution of activity bouts (Figure 5b,e). These results are consistent with those obtained from the experiments with *fmn* mutants.

**Figure 6:**
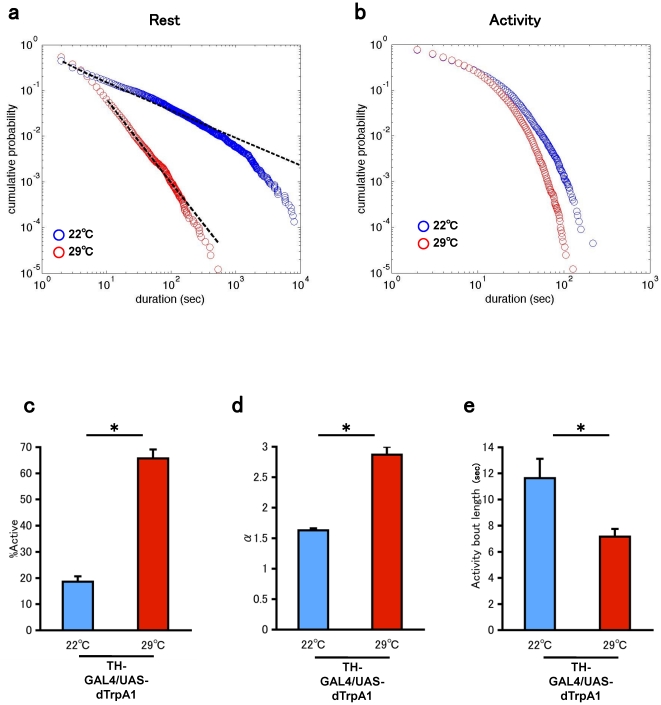
Effect of transient dopamine neuron activation. a. Double logarithmic plots of the cumulative probability of the rest bout for the combined data of TH-GAL4/UAS-dTrpA1 flies(n = 10). blue: 22°C. red: 29°C. b. Double logarithmic plots of the cumulative probability of the activity bout for the combined data of TH-GAL4/UAS-dTrpA1 flies(n = 10). blue: 22°C. red: 29°C. c. The ratio of the total duration of the activity episodes. d. Power-law exponent of the fitted distribution of rest bouts. e. Mean activity bout length. Bars and error bars represent the mean and s.e.m., respectively. Asterisks indicate statistically significant difference (p<0.05 Student's *t* test).

To further study the effect of dopamine, we transiently controlled the firing of dopamine neurons. We expressed the dTrpA1 transgene in the dopamine neurons using the GAL4-UAS system [31]. dTrpA1 is a member of the transient receptor potential cation channel family with highly temperature-dependent conductance, and it participates in the temperature preference behavior both in the larva and adult flies [32], [33]. Neurons, which constitutively express dTrpA1, fire tonically in response to modest heat (>26°C) with little adaptation [34]. Flies carrying both TH-GAL4 and UAS-dTrpA1 were first assayed at 22°C. After recording the activity at 22°C for 24 h, we raised the ambient temperature to 29°C and recorded the activity for further 24 h. Activation of the dopamine neurons realized at high temperature in this assay significantly increased the ratio of activity (Figure 6c), principally through the modulation of the power-law exponent of the distribution of rest bouts (Figure 6a,d). The activation of dopamine neurons did not increase activity bouts but rather paradoxically decreased activity bouts (Figure 6b,e). These experiments were conducted using the same individuals. Therefore, we concluded that the transient activation of dopamine neurons is sufficient for inducing a change in the temporal structure of the rest and activity episodes. These results are consistent with those obtained from the *fmn* mutant and pharmacological analysis.

### Comparison with the DAM system

In our previous study that was performed using the DAM system, we found that the mean activity bout was longer in *fmn* mutant flies than in control flies [18]. The discrepancy between the previous and the present results may be attributed to the difference in the analysis methods. The DAM system is reported to overestimate rest bouts compared to the video method [12]. The difference is probably because of the low temporal resolution of the DAM system compared to that of the video system. To confirm this point, we assessed the effect of bin width on the results obtained from the video system. In order to determine the influence of the temporal resolution on cumulative distributions, we also studied the effect at different data resolutions ranging from 1 fr (1 s) to 60 fr (1 min) by summing the amount of movements. Rest and activity was assigned using the same method.

**Figure 7:**
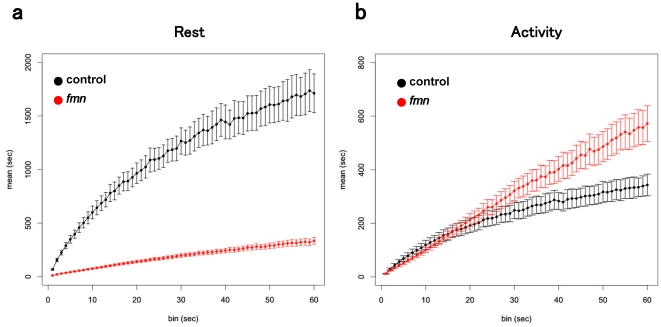
Dependence of the mean bout on the bin width. The degree of movement was summed up in each bin of the specified width. Then, we classified the bins of the specified width to rest or activity using the *k*-means clustering algorithm. The data analyzed in Figure 2a, b were reexamined with different bin widths. a. Mean rest bout for different bin widths. Black: control. Red: *fmn*. b. Mean activity bout for different bin widths. Black: control. Red: *fmn*. Error bars represent s.e.m.

The mean rest bout for control and that for the *fmn* mutants are compared with different bin widths in Figure 7a. As described above, with the smallest bin width of 1 s, the mean rest bout was significantly different between the control and *fmn*. The difference even increased with the bin width because the mean rest bout for the control fly increased dramatically with the bin width, while that of the *fmn* mutant increased gradually.

The mean activity bout did not significantly differ between the control and *fmn* mutant with the bin width of 1 s. The difference remained small up to the bin width of 20 s. When the bin width exceeded 20 s, the mean activity bout for *fmn* became longer than that for the control (Figure 7b). The results with the bin width of 1 min are consistent with those of our previous study performed using the DAM system whose recording period was 1 min [18]. Therefore, the discrepancy between the results obtained with the DAM system and those obtained with the video system is probably caused by the difference in the temporal resolution of the two methods. We also measured the distributions of rest and activity bouts using the DAM system and found an increase in the activity bout in the *fmn* mutant (Figure S4).

## Discussion

We analyzed the temporal organization of rest and activity bouts during locomotor behavior in *Drosophila*. Previous studies on the modulation of fly locomotion mostly used the DAM recording system and focused on gross changes in rest and activity bouts or temporal structure of fly movements on a long timescale as represented by the circadian modulation. To understand the fly's locomotion in more detail, we used a video-monitoring system and obtained the recordings of fly movements over 24 h. Since we wanted to quantify all the movements, we used movement index method rather than movement distance method, which is sensitive in detecting small movements such as grooming during short arousal periods. In addition, the statistical method we employed here, requires a large data set with fine temporal resolution. Because this method compares only two frames next to each other, it allows a long term observation with robustness against noise and high performance calculation for quantification. However, since this method does not take the distance of locomotion into account, it is not suitable for the analysis of the distribution of the distance-based activity like Lévy flight.

The rest bout of the control flies followed the power-law distribution, which is consistent with the results of previous studies [5], [35]. However, commonly used methods for testing the power-law distribution, such as the least square fitting, can generally produce significant systematic errors [9], [20]. Therefore, we applied the statistical methods proposed recently by Clauset et al. and confirmed the power-law nature of the rest bout. On the other hand, the distribution of activity bouts was not power-law. This is the first report which showed the power-law distribution in fly locomotion with a precise statistical method. However, there are still limitations in the analysis since we could not completely rule out log-normal distribution. Thus, further analysis may be required.

Multiple factors, such as circadian rhythm, gender, age, and temperature, modulate the locomotor behavior in *Drosophila* [35]. We studied the effects of the circadian clock and ambient temperature on the temporal organization of rest and activity episodes. *Drosophila melanogaster*, a diurnal organism, shows most locomotor activities during the day and sleep-like behavior during the night [36], [37]. The ratio of activity was significantly higher during the subjective day. However, contrary to our expectation, the distribution of activity bouts was not affected by circadian time. Elevation of the ambient temperature increased the gross amount of activity via a mechanism similar to that of the modulation by the circadian clock. High ambient temperature increased the ratio of activity, but did not increase the activity bouts. Furthermore, the power-law property of the distribution of rest bouts was little affected by the changes in temperature and circadian time. These results suggest that the gross changes in the locomotor activity on a long timescale by circadian rhythm and ambient temperature were predominantly derived from the changes in the number of activity bouts.

In *Drosophila*, dopamine modulates various behavioral aspects from locomotor activities and arousal to learning and memory [18], [19], [38], [39], [40], [41]. As previously reported, an increase in dopamine signaling increased the ratio of activity [18], [19]. In this study, we showed that the power-law property of the distribution of rest bouts was maintained through the modulation of dopamine signaling. Dopamine modulated the power-law exponent of the distribution, which resulted in the alteration of the gross amount of rest over a long timescale. In contrast to the effect on rest bouts, the alteration of dopamine signaling did not affect the activity bouts. This was incompatible with our previous study using DAM system, in which the average length of activity was longer in DAT-deficient *fmn* mutant [18]. This inconsistency was due to the limitation of DAM system, in which short rest periods separating activities could not be detected, as described above for the Figure S4. This is one of advantage of the video system.

Many current models of animal behavior are based on the Poisson process, in which a behavioral event occurs with probability *qdt*, where *dt* is a small time interval, and *q* is the intensity of the event, which is independent of the time and history. The Poisson process results in the exponentially distributed interevent interval (i.e., bout). In our study, the rest bout was found to obey the heavy-tailed distribution. This non-Poissonian nature of the rest bout indicated that the transition rate from rest to activity is not constant in time and regulated by some additional mechanisms, which may be history-dependent. Moreover, this non-Poissonian transition is modulated by factors such as circadian rhythm, temperature, and dopamine signaling. On the other hand, the distribution of activity bouts showed exponential distribution, indicating that some biological process which is constant in time regulates transition rate from activity to rest.

Ellipsoid body, a neuropil structure in the central nervous system of flies, contributes to the power-law property in waiting intervals between walking episodes [42]. Furthermore, memory mutants such as *dunce* and *rutabaga* which have impairment in cAMP signaling result in decreased power-law exponents in the power-law distribution during foraging behavior in *Drosophila* [43]. It would therefore be fruitful to investigate whether cAMP signaling in ellipsoid body contributes to the temporal organization or the brain structure important for memory formation such as the mushroom body regulates neural activity of ellipsoid body.

There are several reports on temporal organizations of rest and activity episodes in humans and mammals. In mouse and human, the distributions of the rest and activity bouts obey the power-law distribution and the exponential distribution, respectively [2], [3]. The results for *Drosophila* revealed in the present study are consistent with these previous studies and demonstrated common behavioral patterns between mammals and invertebrates.

In contrast, in other reports based on the electrophysiological determination of sleep and wakefulness during night time sleep, the sleep bout followed the exponential distribution, and the wake bout follows the power-law distribution [44], [45], [46]. There is also a sleep-like state in *Drosophila melanogaster*, and the sleep is defined as a minimum of 5 min of rest, on the basis of the changes in the arousal threshold [37], [47], [48]. In our study, the power-law property of the rest bout was mainly observed within seconds to a couple of minutes. This may indicate a qualitative difference between sleep and rest. It may also be worthwhile to note that the flexion point (dissociation from the power-law distribution) in the rest bout distribution shifted with some manipulation. In addition, as shown in Figure S4, using conventional DAM system, a majority of time are classified into sleep (more than 70% in control flies), while there were only fractional rest period longer than 5 min in the present study (see Fig. 2). The method we employed here detects small movements and thus eliminated most of the sleep defined by the conventional definition. Thus we need to examine the biological and physiological meaning of these small movements, which were not detected in DAM system, in order to proceed sleep research using the present method.

Finally, it has been reported that the patients with major depression show the decrease of scaling exponent of power-law distribution in resting periods [3]. In mammals, some sleep disorders, including narcolepsy, show the state of instability (hypersomnia and sleep fragmentation) during sleep and wakefulness [49]. The examination of the genetic basis underlying the temporal organization of rest and activity may contribute to the understanding of neuropsychiatry disease such as depression or sleep disorders.

## Materials and Methods

### Fly stocks and drug treatment

Flies were reared on a conventional corn meal, yeast, and glucose agar medium at 25°C as described previously [50]. The following transgenic lines and mutants were used: *fmn* [18], TH-GAL4 [51], UAS-dTrpA1 [33]. To remove possible modifiers and allow comparisons in a common genetic background, we outcrossed all the alleles into the *w^1118^* background over at least 5 consecutive generations. The *w^1118^* stock used for backcrossing was provided by M. Saitoe (Tokyo Metropolitan Institute for Neuroscience, Tokyo, Japan). As controls we used the *w^1118^* flies.

For the pharmacological manipulations, 5% sucrose and 2% agar was used as food. 3-Iodo-tyrosine (3 mM) was obtained from Sigma (St. Louis, MO).

The number of flies used for each experimental condition is 10 except for DAM analysis.

### Quantification of locomotor activity by the video tracking method

All assays were conducted using 2- to 5-d-old flies. Individual flies were housed in a custom-made circular chamber of 2.5 cm diameter and 2 mm height. Briefly, a square black acrylic plate (15×17 cm, 2-mm thick) with 20 holes (diameter, 2.5 cm) in a 4×5 array was placed over food in a square dish (16×18 cm). After a single fly was placed in each hole, a transparent acrylic plate (13×15 cm, 5-mm thick) was placed over it. Flies could walk around and consume food. To record the movements of the flies, an infrared camera was mounted at 14 cm above the chamber. Flies were acclimated to the chamber for at least 24 h at 25°C with a 12 h light/dark (LD) cycle, and then subjected to constant darkness (DD). Images were captured using a custom-made software written in Labview (National Instruments) for 24 h under continuous darkness with an illumination by infrared LEDs. The movement of 20 flies was simultaneously videotaped. The video data was compressed in an mpeg4 format and stored in an Audio Video Interleave (AVI) file. The rate of image capture was 1 frame per second, and the image size was 640×480 pixels. For the neural activation experiment with dTrpA1 transgene, the ambient temperature was first set to 22°C and switched to 29°C at circadian time (CT) 0.

A custom-made software written in Labview was used for analyzing the video images as illustrated in Figure 1a. To quantify the activity of each fly, we set the regions of interest (ROIs) to the area of each chamber. All the images were converted into 8-bit (256-step) grayscale images. We subtracted 2 consecutive images pixel-wise to generate the difference images. A Gaussian filter was applied to the difference image to reduce noise. For the correction of background intensity changes due to the flicker of the infrared illumination, each pixel in the difference image was binarized based on a predetermined threshold. The threshold was set to 20, where the maximum level on the grayscale was 256. This threshold value was sufficiently small compared with the difference achieved by actual fly movements, which is usually above 100. The ratio (%) of suprathreshold pixels in the total number of pixels in ROIs was used as an indicator of the degree of movement (Figure 1b).

### Data analysis

On the basis of the values of the indicator, which obey a bimodal distribution as shown in Fig. 1c, we divided the values into the following 2 classes: rest and activity. In order to classify, we calculated the threshold value of the movement indicator of each fly by the *k*-means clustering algorithm using software R [52]. Then, each 1 sec bin was assigned to rest or activity, and a rest (activity) episode was defined by a series of consecutive rest (activity) bins; rest episodes and activity episodes necessarily alternate. The length of the rest (activity) episode is defined as the rest (activity) bout (Figure 1d).

Rest and activity bouts were statistically analyzed by R. To quantify the distribution of bouts, we calculated the complementary cumulative probability distribution of bouts, 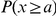, where 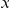 is the variable representing the bout, and 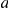 is the threshold. 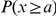 represents the fraction of rest (activity) episode whose length is larger than 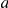 (s). 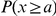 for rest and activity bouts was calculated separately. The cumulative distributions were related to the probability density function 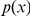, that is, the proportionate fraction of bouts that were approximately equal to 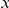, via 
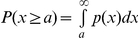
. We will be concerned with whether 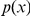 and hence 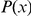 are long-tailed. Cumulative distributions of rest and activity bouts were calculated on the basis of the data from individual flies and those from all the flies combined. We fitted a power-law 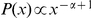 to the cumulative distribution of rest or activity bouts using the maximum likelihood method that carefully estimates the lower bound of 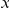, as well as 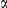 [20]. Assuming that our data are drawn from a distribution that follows a power-law, we can derive maximum likelihood estimators. The data are most likely to have been generated by the model with parameter that maximizes this function.

Next, we performed a goodness-of-fit test to determine the plausibility of the power-law hypothesis. To this end, we generated 1000 surrogate data sets using nonparametric bootstrap method [53], each of which is a set of artificial bouts sampled from the estimated power-law distribution. To calculate the deviation of a distribution, either empirical or artificial, from the estimated power-law distribution, we used the Kolmogorov-Smirnov (KS) statistics, which is the maximum distance between the cumulative distributions of the two distributions to be compared. The resulting *p*-value was defined as the fraction of surrogate realizations such that the KS distance between the distribution generated from a surrogate data set and the estimated power law is larger than the KS distance between the empirical distribution and the estimated power law. Therefore, a large *p*-value implies that the power-law distribution reasonably fits the original data. Note that the notion of *p*-value introduced here is different from the standard one in which a smaller *p*-value in the goodness-of-fit test indicates more significance. To avoid confusion, we refer to the notion of *p*-value introduced here as *p′-*value.

Furthermore, to test the plausibility of the power-law hypothesis as compared to alternative distributions without long tails, we performed the likelihood ratio test [20]. The likelihood ratio is defined as the ratio of the likelihood of the data under the estimated power law to that under an alternative distribution estimated by the maximum likelihood method. If this value is large positive, the power-law assumption is considered to be plausible as compared to the alternative distribution. As alternative distributions, we chose a distribution without long tails, i.e., the exponential distribution 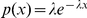 and a distribution with long tails, i.e., the log-normal distribution 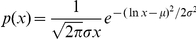.

### Locomotor activity analysis using the DAM system

By using the DAM system (Trikinetics, Waltham, MA), we monitored the activities of the flies by recording infrared beam crossings of individual flies housed in glass tubes (length, 6.5 cm; internal diameter, 3 mm) containing the medium (5% sucrose, 2% agar) as food at one end of the tube. Events were scored at 1-min intervals. We used 2- to 5-d-old male flies. Flies were housed for 3 days under a 12-h LD cycle, and the data were collected after the flies were kept under DD conditions for 3 days. Bins without and with beam crossing were defined as rest and activity, respectively. The definitions of rest and activity bouts for the DAM system are the same as that for the video system.

## Supporting Information

Figure S1 **Distributions of rest and activity bouts during** ***Drosophila*** **locomotor activities.** a. Double logarithmic plots of the cumulative distribution for individual control flies. Broken line represents the cumulative distribution obtained from the combination of different flies and is identical to the distribution shown in Figure 2a. b. Double logarithmic plots of the cumulative distribution for individual control flies. Broken line represents the combined data and is identical to the distribution shown in Figure 2b.
(TIF)

Figure S2 **Effect of size of arena.** a. Double logarithmic plots of the cumulative probability of the rest bout for the combined data of control male flies (n = 10). green: 1 cm blue: 2.5 cm. red: 5 cm. b. Double logarithmic plots of the cumulative probability of the activity bout for the combined data of control male flies (n = 10). green: 1 cm blue: 2.5 cm. red: 5 cm. c. The ratio of the total duration of the activity episodes. d. Power-law exponent of the fitted distribution of rest bouts. e. Mean activity bout length. Bars and error bars represent the mean and s.e.m., respectively. ns, not significant (Tukey–Kramer HSD test).
(TIF)

Figure S3 **Effect of circadian rhythm.** a. 24-h recording of the degree of movement of a control fly. b. 24-h recording of the degree of movement of a *tim^01^* fly. c. Double logarithmic plots of the cumulative probability of the rest bout for the combined data of *tim^01^* male flies (n = 12). b. Double logarithmic plots of the cumulative probability of the activity bout for the combined data of *tim^01^* male flies (n = 12).
(TIF)

Figure S4 a,b Distributions of rest and activity bouts for the data recorded with the DAM system. The bin width is equal to 1 min (n = 64). c, Average total daily sleep for control and *fmn* populations. Sleep is defined as a minimum of 5 min of rest.
(TIF)
