# Supplementary figures and images for: Dopamine Modulates the Rest Period Length without Perturbation of Its Power Law Distribution in Drosophila melanogaster
Source: PLoS One. 2012 Feb 16;7(2):e32007. doi: 10.1371/journal.pone.0032007 (PMC3281125; doi:10.1371/journal.pone.0032007)

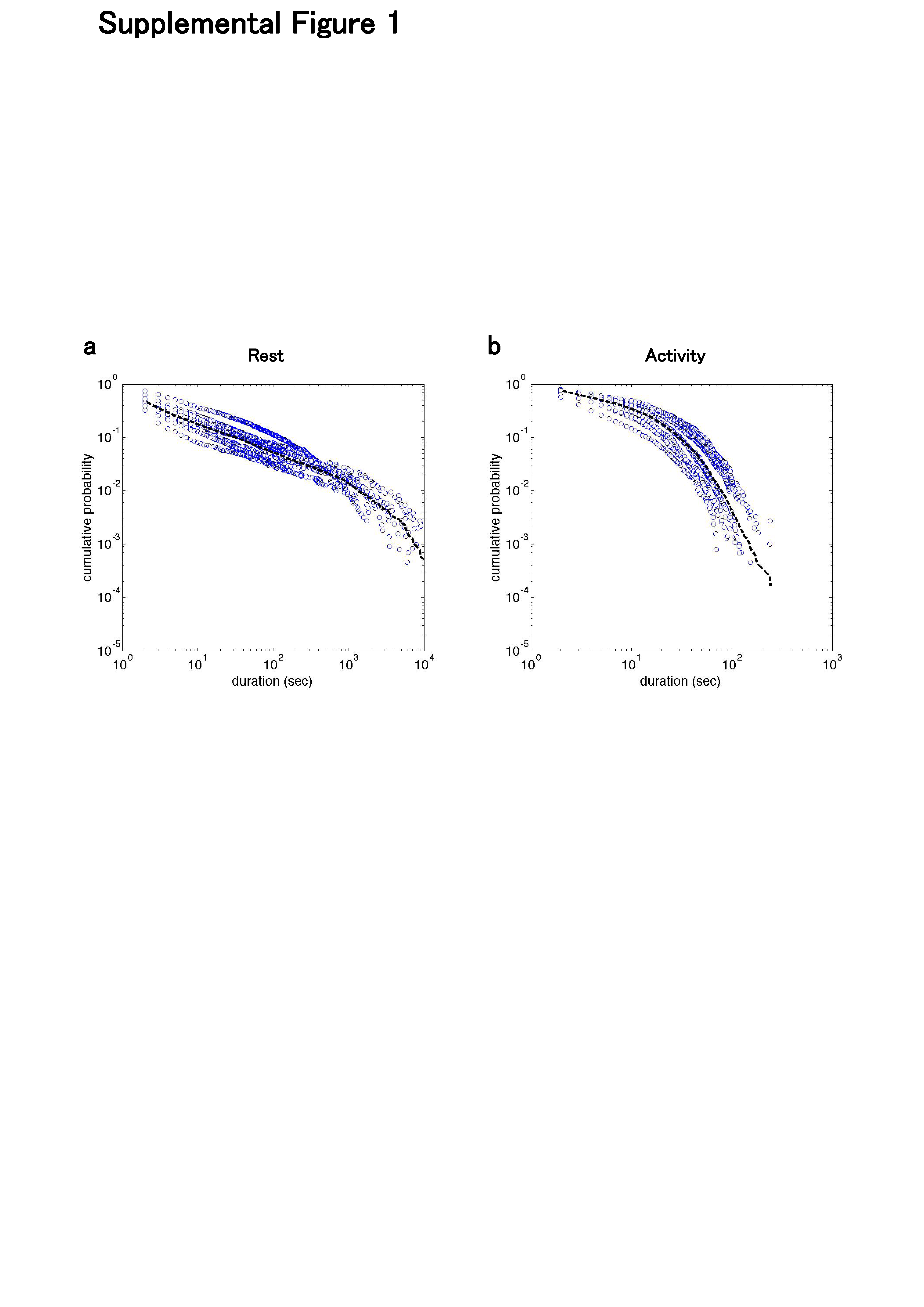

Supplement: Figure S1 — Distributions of rest and activity bouts during Drosophila locomotor activities. a. Double logarithmic plots of the cumulative distribution for individual control flies. Broken line represents the cumulative distribution obtained from the combination of different flies and is identical to the distribution shown in Figure 2a. b. Double logarithmic plots of the cumulative distribution for individual control flies. Broken line represents the combined data and is identical to the distribution shown in Figure 2b. (TIF) [file pone.0032007.s001.tif]

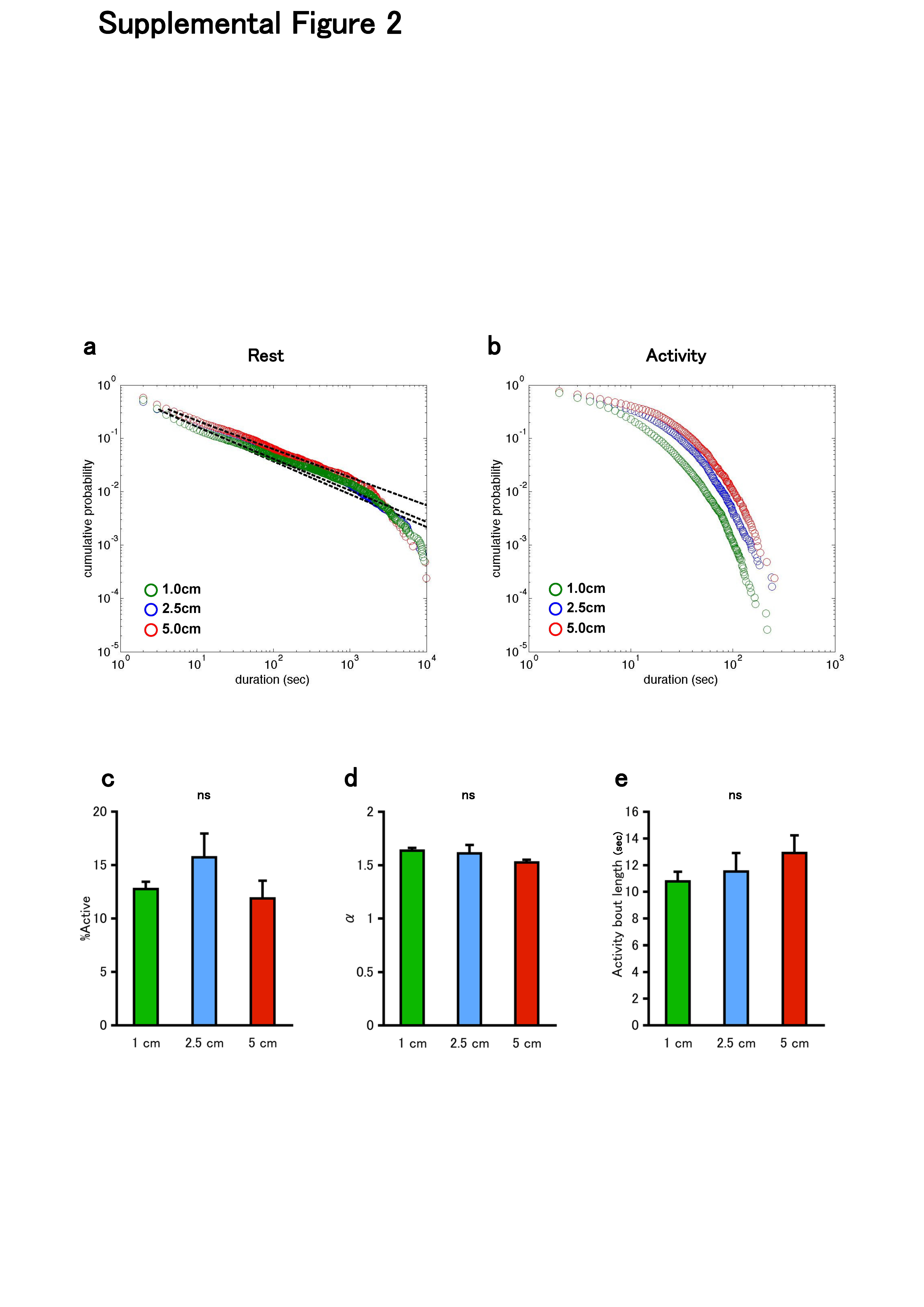

Supplement: Figure S2 — Effect of size of arena. a. Double logarithmic plots of the cumulative probability of the rest bout for the combined data of control male flies (n = 10). green: 1 cm blue: 2.5 cm. red: 5 cm. b. Double logarithmic plots of the cumulative probability of the activity bout for the combined data of control male flies (n = 10). green: 1 cm blue: 2.5 cm. red: 5 cm. c. The ratio of the total duration of the activity episodes. d. Power-law exponent of the fitted distribution of rest bouts. e. Mean activity bout length. Bars and error bars represent the mean and s.e.m., respectively. ns, not significant (Tukey–Kramer HSD test). (TIF) [file pone.0032007.s002.tif]

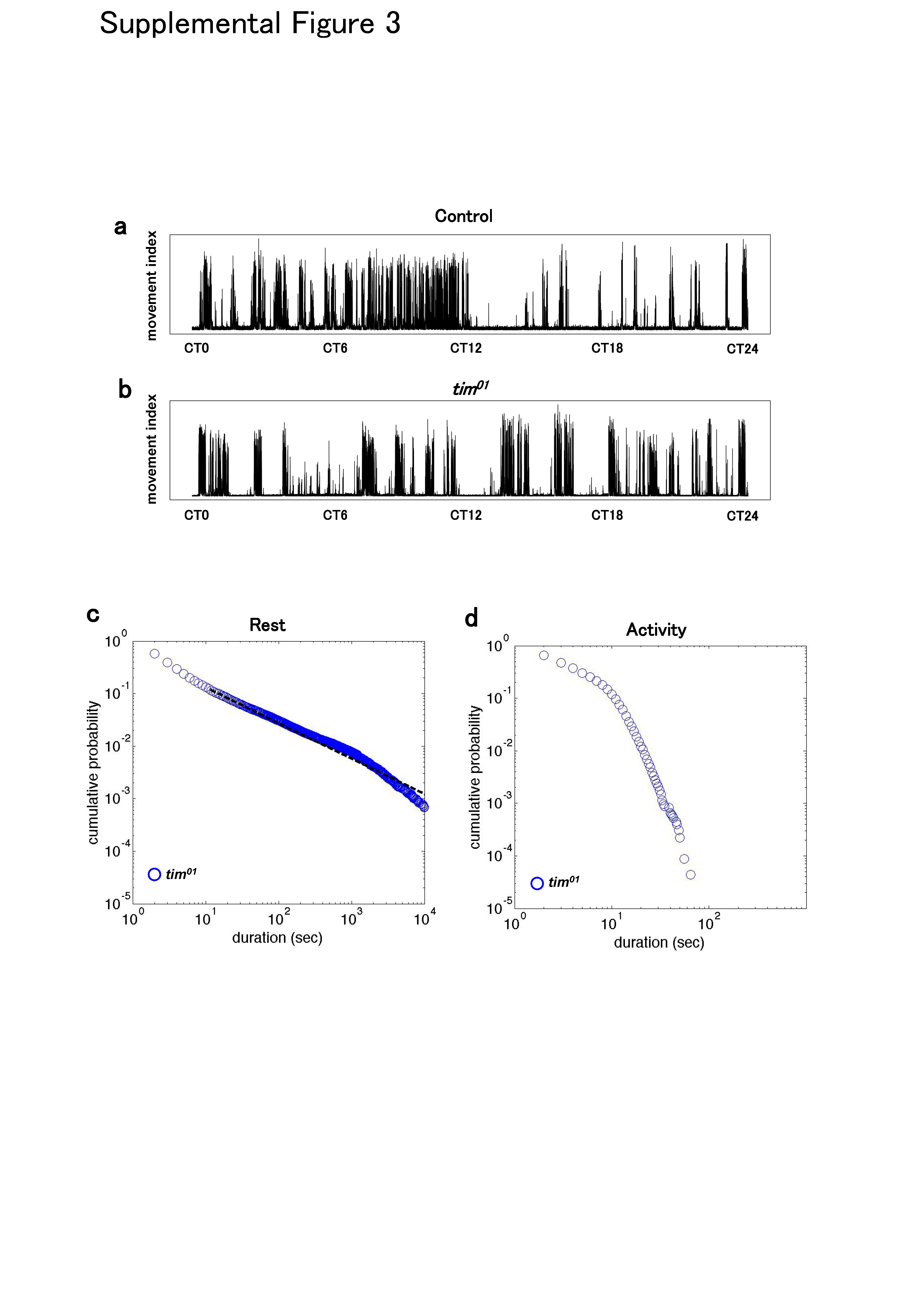

Supplement: Figure S3 — Effect of circadian rhythm. a. 24-h recording of the degree of movement of a control fly. b. 24-h recording of the degree of movement of a tim01 fly. c. Double logarithmic plots of the cumulative probability of the rest bout for the combined data of tim01 male flies (n = 12). b. Double logarithmic plots of the cumulative probability of the activity bout for the combined data of tim01 male flies (n = 12). (TIF) [file pone.0032007.s003.tif]

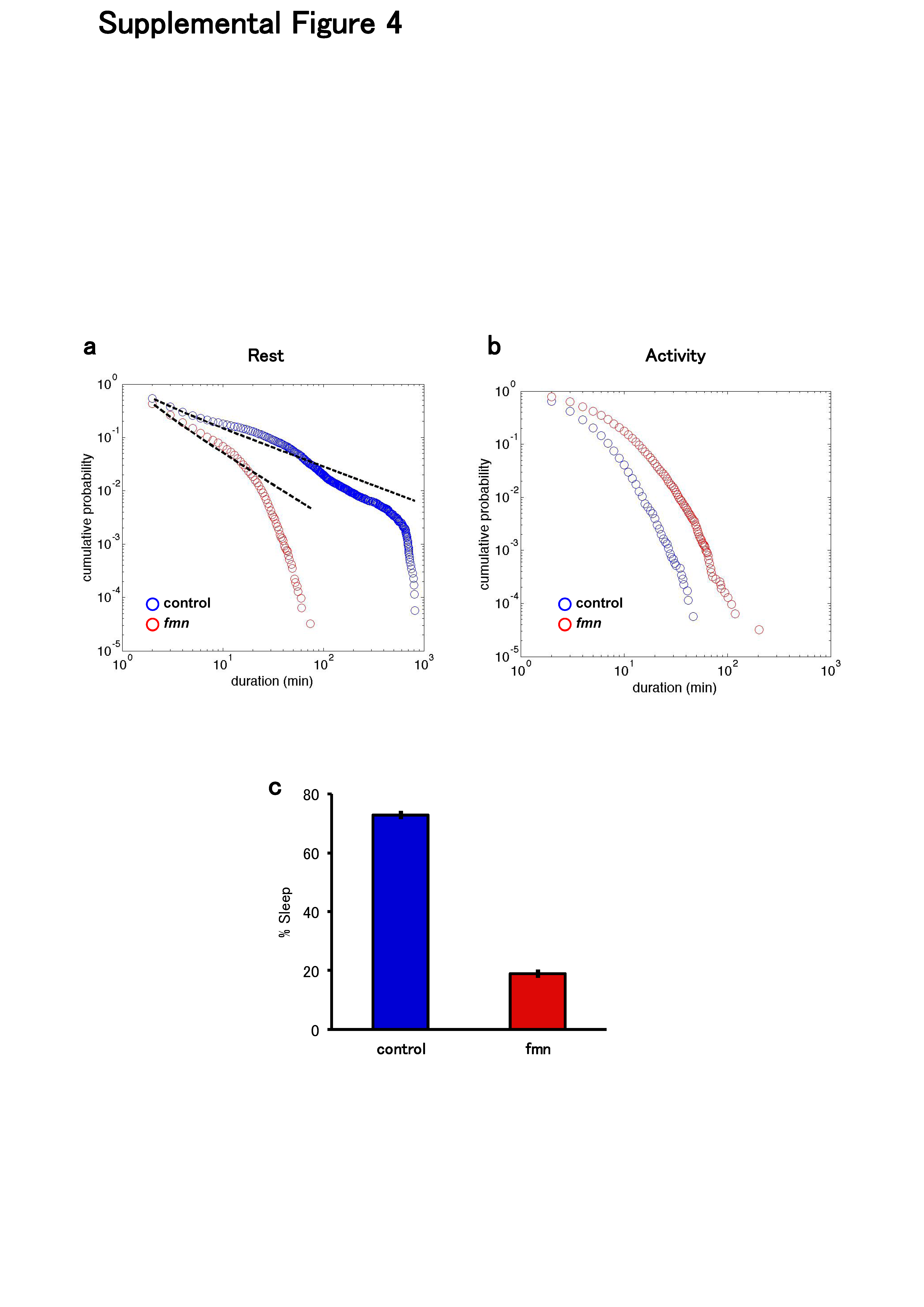

Supplement: Figure S4 — a,b Distributions of rest and activity bouts for the data recorded with the DAM system. The bin width is equal to 1 min (n = 64). c, Average total daily sleep for control and fmn populations. Sleep is defined as a minimum of 5 min of rest. (TIF) [file pone.0032007.s004.tif]
